# Effect of Sodium Trimetaphosphate on Chitosan-Methylcellulose Composite Films: Physicochemical Properties and Food Packaging Application

**DOI:** 10.3390/polym11020368

**Published:** 2019-02-20

**Authors:** Hongxia Wang, Yu Liao, Ailiang Wu, Bing Li, Jun Qian, Fuyuan Ding

**Affiliations:** School of Printing and Packaging, Wuhan University, Wuhan 430072, China; sxcwhx@163.com (H.W.); lyucn@whu.edu.cn (Y.L.); wal@whu.edu.cn (A.W.); libingwhu@163.com (B.L.)

**Keywords:** sodium trimetaphosphate, chitosan/methylcellulose composite film, antibacterial activity, physicochemical property, food packaging

## Abstract

Environmentally friendly food packaging currently attracts much interest. Sodium trimetaphosphate (STMP) finds specialized applications in food, but it is rarely used as a crosslinking agent. In this study, STMP was used as a crosslinking agent to prepare chitosan/methylcellulose composite films. Both antibacterial and physicochemical properties of the composite film were improved by crosslinking with STMP. The crosslinked films, with good antibacterial activity (~99%), had increased tensile strength, a higher elongation at break, a lower swelling ratio and solubility, and a lower enzymatic degradation than the non-crosslinked films. Furthermore, the crosslinked films showed an excellent preservative effect on fresh-cut wax gourd after three days at room temperature. The obtained films crosslinked by STMP can be potentially applied to the food industry, such as food functional packaging, providing a novel alternative to traditional plastic packages.

## 1. Introduction

Globally, the packaging market has witnessed constant growth and plastic materials stand out (accounting for almost 47.65% of materials in value) [1]. However, many environmental concerns arise from these petrochemical plastics that are non-biodegradable and have a low recovery rate. The appeal of biodegradable food packaging films has gradually grown among researchers [2]. Many polysaccharides including starch, cellulose, and chitin have undergone extensive investigation [3,4,5]. Chitosan is available from fungi, crustaceans, and plants [6,7]. As the second most abundant polysaccharide in nature [8,9], it is regarded as an eco-friendly material with advantages including safety, nontoxicity, edibility, biodegradability, and antimicrobial properties [10,11,12,13]. The cationic property (–NH_3_^+^) of chitosan permits it to establish electrostatic interactions with other compounds, and confers antibacterial properties to composite films [14,15].

Methylcellulose is also a potential material for the production of edible composite films, due to its biodegradability, large availability, and low cost [16]. It has excellent film-forming properties and its film possesses good mechanical properties [17]. Moreover, it is promising as a functional material to modify chitosan and has good compatibility and miscibility for chitosan [18]. However, the practical application of pure chitosan film is limited by mechanical properties like brittleness and that of pure methylcellulose film is limited by the lack of antimicrobial properties, which impose restrictions on the use of these films at the industrial level. The combination of these polysaccharides and the crosslinking reaction may make up for the deficiencies and meet the requirements of food packaging.

Chemical crosslinking is a feasible way to improve the physical and biological properties of films. Sodium trimetaphosphate, a safe and non-toxic crosslinking agent suitable for polysaccharides to improve their functional properties, has been used in many research studies and reports [19]. For example, it has been used for crosslinking biopolymers such as cellulose [20], xanthan [21], and starch [22,23,24], and can decrease the solubility of proposed materials [25]. However, research carried out to crosslink a composite biopolymer film using sodium trimetaphosphate has been rare.

**Scheme 1:**
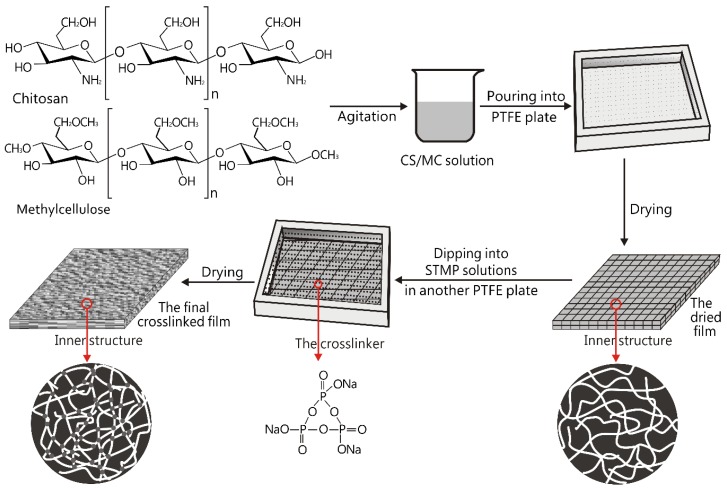
Schematic illustration of the preparation of sodium trimetaphosphate-crosslinked chitosan/methylcellulose composite films.

In this study, sodium trimetaphosphate was used to crosslink the chitosan/methylcellulose composite films. The preparation of the crosslinked composite film is shown in Scheme 1. The chitosan and methylcellulose were first mixed to prepare a dried composite film without crosslinking. Then, the dried composite film was put into a sodium trimetaphosphate solution to induce the crosslinking. The crosslinked composite film was finally obtained after drying. The aim of this study was to investigate the effect of sodium trimetaphosphate on the physicochemical and antibacterial properties of biopolymer-based composite films. In addition, the obtained film was applied to the storage of fresh-cut wax gourd, demonstrating the possible use as functional packaging material for food applications. The significance of this work was to fabricate an antibacterial film based on sodium trimetaphosphate-crosslinked chitosan/methylcellulose and to apply it to food preservation. We believe that this crosslinked composite biopolymer film may have a potential application in food packaging.

## 2. Materials and Methods

### 2.1. Materials

Chitosan was purchased from Shanghai Ruji Biotechnology Development Corporation (Shanghai, China), with a degree of deacetylation of 85% and a *M*_w_ of 750 kDa. Methylcellulose (16000 CPS) was purchased from Zhengzhou Zhuochuang Trading Co. Ltd. (Zhengzhou, China). STMP (Na_3_P_3_O_9_) and lysozyme were bought from Aladdin (Shanghai, China). Acetic acid was bought from Shanghai Reagent Co., Ltd. (Shanghai, China). *Escherichia coli* (*E. coli*), *Staphylococcus aureus* (*S. aureus*), and plate count agar (PCA) were provided by both Wuhan University and the Chinese Academy of Sciences (Wuhan, China).

### 2.2. Fabrication of the Films

The films based on chitosan and methylcellulose were prefabricated by a casting-solvent evaporation technique. Chitosan acetic acid solution (3 wt %, CS) in 2 wt % acetic acid solution and methylcellulose solution (3 wt %, MC) in distilled water were prepared separately and stored in containers. Then, parts of the two solutions were mixed by mechanical agitation at the speed of 1500 r/min for 30 min, after which bubbles were removed by centrifugation for 5 min, finally obtaining chitosan-methylcellulose solutions (CSMC). These solutions were poured into the customized polytetrafluoroethylene (PTFE) plate respectively (with inner groove length 200 mm × width 150 mm × height 5 mm) and were dried for 48 h at 25 ± 0.1 °C with 50% RH (relative humidity). After five days, the dried films were obtained and washed with distilled water to remove the residual acetic acid, followed by drying again. The dried films were then crosslinked in STMP solutions, followed by washing the residual STMP solution with distilled water. These films were dried again for 48 h at 25 ± 0.1 °C with 50% RH. These different samples with different reaction parameters are illustrated in Table 1. Finally, the crosslinked films were naturally dried for 12 h at 25 ± 0.1 °C with 50% RH before characterization and investigation.

### 2.3. Response Surface Methodology

The influence of methylcellulose weight ratio, concentrations of sodium trimetaphosphate, and crosslinking time on the inhibition zone (against *Escherichia coli*) of the developed films was analyzed by response surface methodology (Design-Expert Software, 8.0.6, from Stat-Ease, Inc., Minneapolis, MN, USA). This operation helped to determine the best formulations for the films.

### 2.4. Characterization of Films

To confirm the interaction between chitosan, methylcellulose, and STMP, infrared spectra of these films were recorded using an FTIR5700 Fourier Infrared Spectroscopy Analyzer (Thermo, Waltham, MA, USA) from wavenumber 500 to 4000 cm^−1^, with a resolution of 4 cm^−1^ [26].

An X-ray diffractometer (Xpert Pro, PANalytical B.V., Almelo, Netherlands) was used to perform XRD studies. The patterns were recorded by scanning continuously from 10° to 90° (2θ) at 40 kV and 40 mA.

Thermogravimetric analysis (TGA) tests were performed using a HITACHI thermal analyzer (STA7300, Tokyo, Japan). All the film samples (approximately 8.0 mg) were heated (Pan: AI_2_O_3_) from 25 to 825 °C, at 15 °C min^−1^, under a nitrogen atmosphere.

Scanning electron microscopy (SEM) of films was also recorded. Dried film pieces were placed on the metal stub with double-side conductive tape and coated with a layer of gold (40–50 nm) in vacuum, using a sputter coater. Then, the samples were microscopically examined using SEM (Zeiss SIGMA, Jena, Germany). At least six random positions of each sample were selected for recording [27].

Rheological investigation of film-forming solutions was carried out using a rheometer at 25 ± 0.1 °C with 50% RH. The rheometer (KINEXUS PRO, Malvern, Malvern, UK) was equipped with a parallel plate (diameter 40 mm and geometry serial number PU40 SR2281SS). Shear rate ramp ranged from 0.001 to 10,000 s^−1^ and then back to 0.001 s^−1^. The viscosity behaviors of the film-forming solutions were recorded.

The transparency of the obtained films was investigated using a UV-VIS-NIR spectrophotometer (UV-3600, Shimadzu, Kyoto, Japan) at absorbance values set at 500 nm (A500). The color of these films was measured using an NS820 instrument (Shenzhen Sanenchi Technology Corporation, Shenzhen, China), under illuminant D50 (color temperature: 5000 K). Total color difference ∆E was calculated using Equation (1):

∆E = [(∆L)^2^ + (∆a)^2^ + (∆b)^2^]^0.5^_,_ (1)

where L* indicates white (0) to black (100), a* indicates red (+) to green (−), and b* indicates yellow (+) to blue (−); and where ∆E, ∆L, ∆a, and ∆b represent differences in the color parameters.

A tensile tester (INSTRON, Boston, MA, USA) was used to characterize the tensile strength and elongation at break of the preconditioned films (50 mm × 20 mm) with some modifications, at a constant speed of 0.5 mm s^−1^, under 25 ± 0.1 °C with 50% RH [28]. Bluehill LE software (version 3.69, INSTRON, Boston, MA, USA) associated with the tester recorded the curves of force as a function of deformation. All the measurements were performed in triplicate. Tensile strength (TS) can be calculated using Equation (2) and the elongation at break of films (EAB) can be measured using Equation (3):

TS = F/(L × X),(2)

where F, X, and L indicate axial tensile force, film thickness, and width of the membrane, respectively; and

EAB = (H − H_0_)/H_0_ × 100, (3)

where H_0_ indicates the initial of height of film and H indicates the final height of film after deformation, respectively.

Swelling and solubility. At 25 ± 0.1 °C with 50% RH, all the dried films were cut into 15 mm × 15 mm pieces and accurately weighed. Then, they were placed in a glass vial with 100 mL water. After a specified interval of time, the film samples were taken out, cleaned with soft paper to remove surface water, and reweighed. The swelling ratio was calculated using Equation (4) [1]. The residual samples were dried at 70 °C in a vacuum oven for 24 h and were later weighed again. Film solubility was calculated using Equation (5) [29]. During the test, each sample was tested three times.

SR = (W_S_ − W_D_)/W_D_ × 100, (4)

where SR, W_D_, and W_S_ indicate swelling ratio, weight of the films before dipping in water, and weight of the films after taking them out from the water and cleaning, respectively.

S = (W_D_ − W_R_)/W_D_ × 100, (5)

where S, W_D_, and W_R_ indicate solubility, weight of the dried films before dipping in water, and weight of the dried residual films, respectively.

### 2.5. Antibacterial Activity

The antimicrobial test was performed using *Escherichia coli* (*E. coli*) and *Staphylococcus aureus* (*S. aureus*) which are commonly used in research, according to the report with some modifications [29,30]. The film samples in a bottle were seeded with bacteria-cultured broth (1 mL, 1 × 10^5^ cfu mL^−1^). After the inoculation (24 h) and the normal saline addition, the inoculated medium was plated on nutrient agar and was incubated for 48 h. The inhibition ratio of bacterial growth in these films was calculated using Equation (6). Then, all these films were stored at 25 ± 0.1 °C with 50% RH. After 90 days, these films were taken out for the observation investigation.

IR = (CC − CT)/CC × 100,(6)

where IR, CC, and CT indicate inhibition ratio, bacterial count from the inoculated plates without films, and bacterial count from the inoculated plates with films, respectively.

### 2.6. Enzymatic Degradation 

Lysozyme has been used as a preservative in many foods. The enzymatic degradation of all films (cut into 1.5 cm × 1.5 cm pieces) was performed in phosphate buffer solution (pH = 6.8) containing 10 mg ml^−1^ of lysozyme at 25 ± 0.1 °C with 50% RH. After 90 days, the samples were taken out, cleaned, and weighed. The degradation ratio was calculated using Equation (7). After enzymatic degradation, these films were recorded under SEM. During the test, each sample was tested three times.

DR = (W_B_ − W_A_)/W_B_ × 100, (7)

where DR, W_B_, and W_A_ indicate degradation ratio, the dried weight before degradation, and the dried weight after degradation, respectively.

### 2.7. Application on Fresh-Cut Wax Gourd

Wax gourd was obtained from a market of agricultural products (Wuhan, China). The wax gourd was washed, peeled, and sliced into pieces of approximately 1 cm thickness. These slices were randomly divided into several groups, with the obtained film wrapping up the slices, which was then placed into a valve bag. The control sample was put into the valve bag without the prepared film. After storage for three days, these samples were taken out for observations about dryness, freshness, and color [31].

## 3. Results and Discussion

### 3.1. Response Surface Analysis

Response surface methodology explores the relationships between several explanatory variables and response variables. It was used to help determine the best formulations for crosslinked films. Analysis of variance (ANOVA) helped to obtain models that demonstrated the diameter of the inhibition zone against *Escherichia coli* obtained as a function of MC/(CS + MC), concentration of sodium trimetaphosphate, and crosslinking time in the edible coating (Table 2). The final equation for the diameter of the inhibition zone is as follows:

DIZ = 12.2 − 1.04 × A − 0.037 × B − 0.88 × C + 0.075 × A × B + 0.5 × A × C + 0.1 × B × C − 0.89 × A^2^ − 0.24 × B^2^ − 0.31 × C^2^, (8)

where (DIZ, A, B, and C) = (diameter of inhibition zone, MC/(CS + MC), STMP concentration, and crosslinking time).

**Figure 1:**
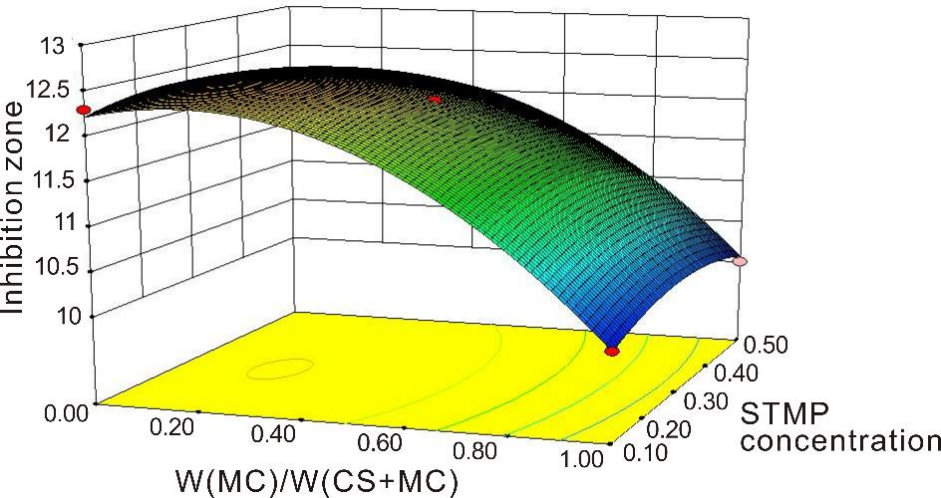
Surface plot (3-D) for thickness. Effect of MC/(CS + MC) and concentration of sodium trimetaphosphate on the diameter of the inhibition zone against *Escherichia coli*.

The model was used to generate the response surface as shown in Figure 1. The maximum diameter of the inhibition zone against *Escherichia coli* was obtained with the MC/(CS + MC) range from 0 to 0.3, the STMP concentration from 0.1% to 0.3 %, and the crosslinking time of 2.5 h, respectively. Thus, we chose the optimized formulation to prepare the films and compared their properties. The MC/(CS + MC) ratios (0.15 and 0.3) were adopted, with the STMP concentration of 0.1% and the crosslinking time of 2.5 h. Chitosan film and chitosan/methylcellulose film (MC/(CS + MC) = 0.15 and 0.3) were coded as CSF, CSMCF1, and CSMCF2, respectively. The crosslinked films based on chitosan, methylcellulose, and chitosan/methylcellulose were coded as CCSF, CCSMCF1, and CCSMCF2, respectively.

### 3.2. Characterization of Films

**Figure 2:**
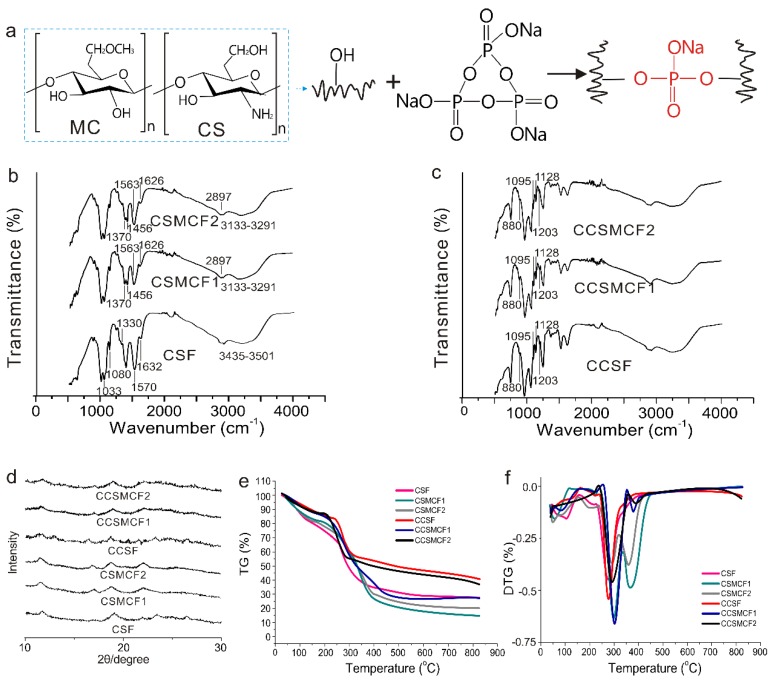
(**a**) The crosslinking reaction between polysaccharides and STMP; (**b**) FTIR spectra of non-crosslinked films; (**c**) FTIR spectra of crosslinked films; (**d**) X-ray diffraction patterns of the prepared films; (**e**) thermograms of the films; (**f**) derivative curves of the films.

The cast films based on chitosan and methylcellulose were crosslinked by sodium trimetaphosphate (Figure 2a). The interaction between heterogeneous polymers can produce some changes to the respective spectra of their components, which has been often characterized by FTIR spectrum. Figure 2b,c displays the FTIR spectra of these prepared films. As for CSF, the peak band from 3435 to 3501 cm^−1^ was attributed to the O–H stretching vibration. The peaks at 1632, 1570, and 1330 cm^−1^ were attributed to Amide I, Amide II, and Amide III, respectively. The peaks at 1632, 1570, and 1330 cm^−1^ were attributed to C=O stretching (amide I), N–H bending (amide II), and C–N stretching vibrations (amide III), respectively [10,26]. The peaks at 1080 and 1033 cm^−1^ were attributed to C–O stretching of chitosan’s saccharide structure [32]. With respect to CSMCF1 and CSMCF2, the peaks at 1370, 1456, and 2897 cm^−1^ were assigned to C–H bend and stretch vibration from methyl groups [33]. The shift in Amide I of chitosan to 1626 cm^−1^ and the shift in Amide II to 1563 cm^−1^ confirmed the interaction between CS and MC. The shift in chitosan O–H vibration to 3133–3291 cm^−1^ may be due to hydrogen-bond interaction between CS and MC. Some studies have reported that the enhanced hydrogen bond could promote the peak to the low wave number [27]. As for these crosslinked films, peaks at 1203, 1128, 1095, and 880 cm^−1^ indicated the phosphate peaks (P=O, –PO_2_, –PO_3_, and P–O–P, respectively), thus confirming the successful crosslinking interaction between the two polysaccharides and sodium trimetaphosphate.

An insight into microstructure is critical for film materials to study further properties like mechanical properties. Figure 2d shows the XRD patterns of the non-crosslinked and crosslinked films. CSF demonstrated two obvious diffraction peaks at 2θ = 12° and 18.9°, which were corresponding to crystal forms I and II, respectively [34]. When dissolved in an acetic acid aqueous solution, the crystal region of raw chitosan was completely destroyed. After drying, the partial reconstitution of chitosan’s crystal region resulted in the crystal difference from the raw chitosan, thus obtaining more amorphous regenerated chitosan [10]. As for CSMCF1 and CSMCF2, the broad peak around 22° could be attributed to the intermolecular hydrogen bonding along with a short-distance order for the sample chains in the methylcellulose polymer [35]. The XRD data demonstrated that the crystal structure of methylcellulose in the films was consistent with the JCPDS file #003–0192 (a file that contains the standard diffraction pattern for methylcellulose), which possessed the cellulose phase [36]. With the presence of chitosan and methylcellulose, the characteristic peaks were visible for both chitosan and methylcellulose, but broader gradually, which may be due to the interaction among CS and MC molecules and the miscibility at the molecular level. As for the crosslinked films (CCSF, CCSMCF1, and CCSMCF2), all peaks became weak, flat, and broad gradually in comparison with the non-crosslinked films (CSF, MCF, and CSMCF), indicating that the crosslinking reaction decreased the crystallinity [18]. The covalent linkage limited the molecular movement of chitosan and methylcellulose and resulted in an amorphous status.

In order to characterize the water retention property and the possible degradation processes of the developed films, thermogravimetric analysis (TGA) was carried out for the determination. Figure 2e–f shows the thermograms and derivative curves of the prepared films. In general, these films possessed different degradation processes and witnessed multi-step thermal decomposition. All films suffered small weight losses around 50–100 °C, which could be attributed to the evaporation of the residual solvent (e.g., water and residual acetic acid) in these films by casting method [37]. CSF had the maximum decomposition and a sharp weight loss around 275 °C due to thermal scission of the polymer backbone, which was in accordance with reported decomposition values from 200 to 300 °C [10]. The maximum decomposition of methylcellulose was reported at approximately 360 to 380 °C. CSMCF1 and CSMCF2 had two distinct peaks of maximum decomposition at approximately 290 and 360 °C in the degradation of the composite films, which respectively corresponded to chitosan and methylcellulose. The incorporation of methylcellulose increased the thermal stability by 15 °C, which was probably due to the interaction between chitosan and methylcellulose. As for the crosslinked films, STMP had a slight effect on the thermal stability of the films. Overall, the films with methylcellulose had a higher maximum decomposition temperature than the other films, due to the interaction among polysaccharides. 

**Figure 3:**
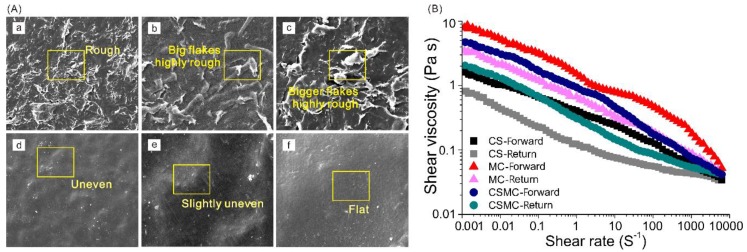
(**A**) SEM micrographs of the prepared films. a, CSF; b, CSMCF1; c, CSMCF2; d, CCSF; e, CCSMCF1; f, CCSMCF2. The scale bar is 500 μm. (**B**) Shear viscosity of the CS, MC, and CSMC solutions.

The molecular structures of methylcellulose and chitosan are very similar and thus the blended films are expected to have excellent compatibility, miscibility, and affinity. SEM analysis characterized the films’ morphology. The films’ microstructure obtained by SEM offered detailed information on the microcosmic arrangement and promoted the study of their structural properties. As shown in Figure 3A a–f, all films possessed a rough surface with uneven morphologies under SEM. In comparison, the surface of films with methylcellulose was relatively slightly rougher (in Figure 3A a–c), which was probably due to the higher viscosity of film-forming solutions containing methylcellulose (in Figure 3B) [27,38]. Figure 3A d–f shows the surface morphologies of the films crosslinked by sodium trimetaphosphate. The three crosslinked films became more compact and uniform. The phenomenon was attributed to the chemical crosslinking through sodium trimetaphosphate. CCSMCF2 was the smoothest and appeared to be the most suitable and satisfying. The interaction between chitosan and methylcellulose and chemical crosslinking by sodium trimetaphosphate may jointly contribute to the compact, flat, and smooth film. 

The visual appearance of the obtained films could influence consumer preferences and acceptance. Table 3 shows the UV absorbance and CIE color parameters of these films. There is significant difference at A500 (the UV absorbance at 500 nm) values and CIE color parameters of the non-crosslinked films and crosslinked films. As for the non-crosslinked films, CSF had the lowest A500 value (0.611) and the CSMCF2 had the highest A500 value (5.081) with the A500 value of CSMCF1 being medium, which could be attributed to the fact that the addition of methylcellulose affected the transparency. Overall, the crosslinked films had higher A500 values, which was probably due to the introduction of sodium trimetaphosphate and the compact surface formed by the crosslinking between polysaccharides and sodium trimetaphosphate. The obtained films differed significantly in color values, demonstrating the significant color differences among these films. L* and ∆E values demonstrated that CCSMCF2 was much whiter than CSF and had the largest color difference. In addition, the high ∆E values of the crosslinked films indicated that the crosslinking reaction had a certain effect on the color differences. In general, the crosslinking reaction produced some changes in the transparency and color of the films.

**Figure 4:**
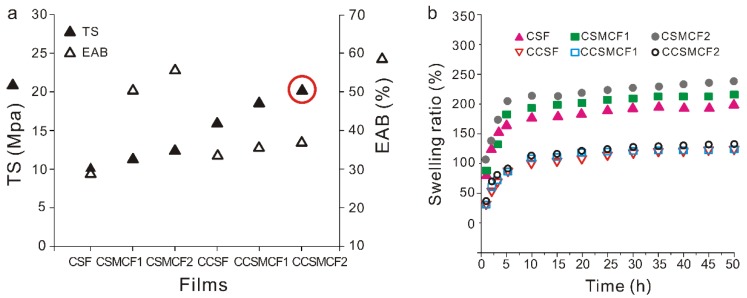
(**a**) The mechanical properties of the prepared films; (**b**) the swelling ratio of the obtained films.

Mechanical properties, an important index for film application, were investigated, including the tensile strength (TS) and the elongation at break (EAB) of the non-crosslinked and crosslinked films. These two parameters represent the maximum strength for the film while withstanding applied tensile stress and maintaining structural integrity and the ability of the film to stretch for easy handling, respectively [34,38]. As shown in Figure 4a, all the prepared films displayed different mechanical behavior patterns under the tensile tester. The relative standard deviation was lower than 5%. TS and EAB values of CSF were 10 MPa and 29%, respectively. The incorporation of methylcellulose improved the mechanical properties of chitosan-based films, which was demonstrated in CSMCF1 and CSMCF2 with higher values (TS and EAB) than CSF. On the other hand, the crosslinking reaction had a great impact on the mechanical properties of these films. Crosslinking significantly increased the mechanical strength of the films. EAB values of the crosslinked films were reduced and this phenomenon indicated the lower flexibility, which may be attributed to the decreased chain mobility of polymer molecules because of the aforementioned strong interaction. Moreover, all TS values of the crosslinked films were higher than that of the non-crosslinked films. This was probably due to the fact that the strong interaction (e.g., covalent bond) between the polymeric matrix and the sodium trimetaphosphate enhanced the strength of the polymer. These results were similar to those obtained in other reports [29]. In comparison with some methylcellulose-based films in the reported literature [39,40,41], the EAB values of the obtained crosslinked films were much higher and more satisfying.

Swelling and solubility, two important properties of films, were adopted to characterize the water absorption capacity and water resistance of the films, especially in a humid environment. As shown in Figure 4b and Table 4, CSF was gradually swelled, with a final swelling ratio of approximately 196% and a low solubility of 1.5%, because chitosan molecules are not soluble in water. Compared with CSF, CSMCF2 experienced an intermediate swelling ratio (up to 239%) and a higher solubility of 23%, due to the strong hydrophilicity of methylcellulose molecules. Although close interactions between chitosan molecules and methylcellulose molecules occurred, the interaction could only hinder part of the interaction between the –OH groups of methylcellulose and water molecules. Methylcellulose molecules on the film surface came into contact with water, leading to the high swelling ratio and solubility.

After crosslinking, some changes to swelling and solubility occurred in different films. As for CCSF, the film had a lower final swelling ratio of 139%, with a lower solubility of 1%. The crosslinking reduced the number of hydrogen bonds to water [1]. In addition, it resulted in the compact surface and prevented the water from entering the inner structure, thus obtaining lower swelling. What is more, with respect to CCSMCF1 and CCSMCF2 that contained methylcellulose, the swelling and solubility of the crosslinked films were much lower than the original non-crosslinked films, demonstrating that the hydrophilicity of these films was decreased. The reason may be that (1) the methylcellulose molecules on the films surface were crosslinked by sodium trimetaphosphate, thus lowering the number of molecules dissolving into water, (2) the crosslinking process by sodium trimetaphosphate on the surface led to the stronger and more compact film, and decreased the polymer relaxation and diffusion of water into the polymer, thus lessening the swelling and solubility, and (3) the flexibility and mobility of polymer chains were reduced, thereby resulting in the decrease of swelling and solubility [28]. Higher water solubility would limit the film’s applications to food with high water content. On the other hand, it could promote biodegradability of the film. Compared with other reported chitosan- or methylcellulose-based films, the crosslinked films showed lower swelling ratios [29] and lower solubility [1,42]. Overall, the crosslinked films were more satisfying, due to the low final swelling ratio and low solubility.

### 3.3. Antibacterial Activity

Antibacterial activity will increase the additional value of the films’ application to food. Table 4 shows the antibacterial properties of the prepared films. Chitosan has strong antimicrobial activities against a wide range of microorganisms, due to the protonation of –NH_2_, the chelation function inside and outside the cell membrane, the adsorption effect on the surface of cell, the damage on the membrane, and the activation on the chitinase for further damage on the cell wall. In general, the effects on *E. coli* and *S. aureus* were almost the same for each film sample (see Table 4). As expected, it was observed that CSF showed a high inhibition ratio of the growth of *E. coli* and *S. aureus* (99.8% and 99.9 %, respectively). As for CSMCF1 and CSMCF2, the inhibition ratios of bacterial growth were also high, but slightly lower than the one for CSF. The reason could be attributed to the addition of methylcellulose, which has no antimicrobial activity. In addition, the interaction between chitosan molecules and methylcellulose molecules influenced the inhibition efficiency by reducing the interaction between chitosan molecules and bacteria. With respect to the crosslinked films, CCSF, CCSMCF1 and CCSMCF2 showed a slightly lower inhibition efficiency than the non-crosslinked films. Sodium trimetaphosphate has no antibacterial property. It crosslinked with chitosan molecules and formed compact structures, which hindered the mobility of other groups like –NH3^+^. The crosslinked films showed a higher inhibition efficiency than that of another reported methylcellulose composited film (93.6 %) [29].

**Figure 5:**
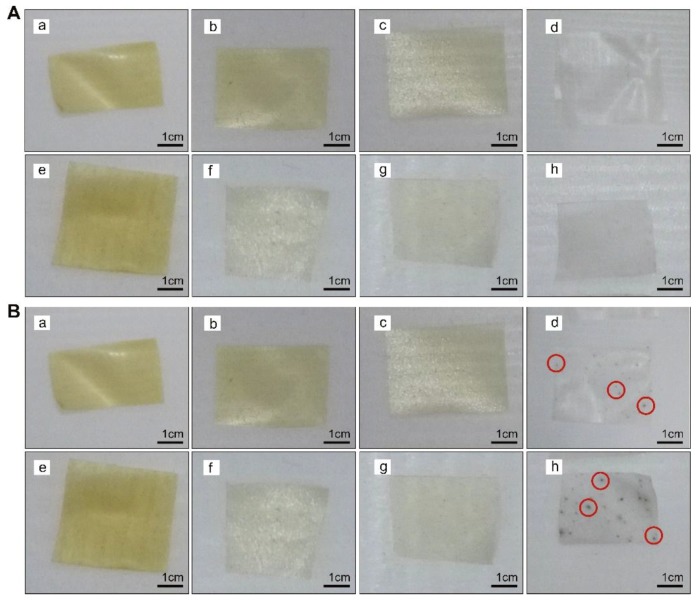
The films (**A**) before and (**B**) after 90-day storage. a, CSF; b, CSMCF1; c, CSMCF2; d, MCF; e, CCSF; f, CCSMCF1; g, CCSMCF2; h, CMCF.

As shown in Figure 5, some changes to the films’ appearance occurred, before and after the 90-day storage. As for the films MCF (methylcellulose film) and CMCF (crosslinked methylcellulose film), some black spots appeared which were microorganism colonies. When these films were dried naturally in the surrounding environment, the microorganisms in the air stayed on the films and grew gradually, thereby forming these microorganism colonies. As for the films containing chitosan (CSF, CSMCF1, CSMCF2, CCSF, CCSMCF1, and CCSMCF2), the changes were slight, because of chitosan’s excellent antibacterial activity.

### 3.4. Enzymatic Degradation

**Figure 6:**
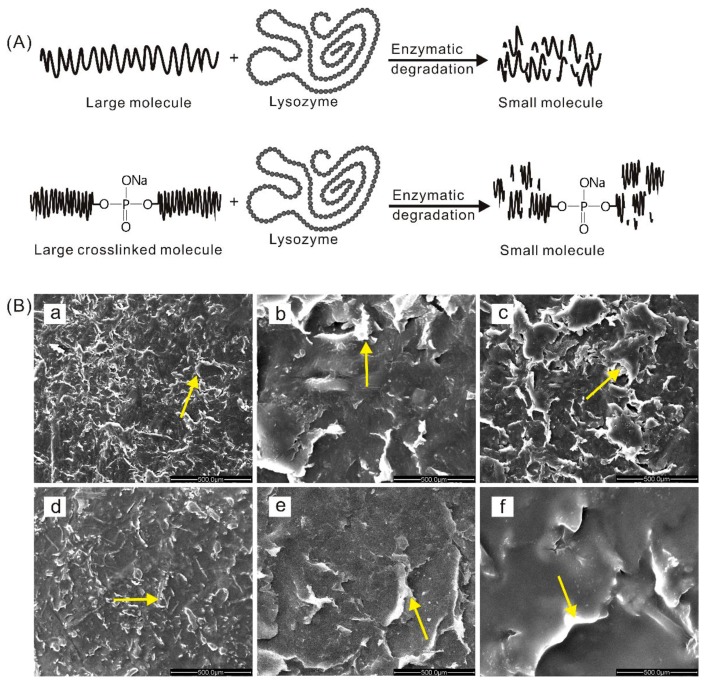
(**A**) The degradation of molecules under lysozyme; (**B**) SEM of films after degradation. a, CSF; b, CSMCF1; c, CSMCF2; d, CCSF; e, CCSMCF1; f, CCSMCF2.

Lysozyme, non-toxic and non-side-effect, has been used as a natural food preservative. It is now widely used in aquatic products, meat, cakes, etc. In addition, it can destroy the β-1,4 glucosidic bond in chitosan and decompose it into smaller molecules, thus breaking the chain and degrading the film (Figure 6A). From Table 4, it is observed that CSF degraded over 90 days and had a high degradation ratio (up to 2%). CSMCF1 and CSMCF2 also had a high weight loss (up to 6%), which could be caused by lysozyme degradation and solubility in water. After crosslinking, CCSF had a lower degradation ratio (1.8%), which could be attributed to the compact films formed by sodium trimetaphosphate crosslinking. The compact films prevented the lysozyme molecules from entering into the inner structure and protected the inner chitosan molecules against degradation. Moreover, because of the crosslinking on the films’ surface, fewer chitosan molecules were exposed to lysozyme, thus reducing the degradation. CCSMCF1 and CCSCF2 also had lower weight losses (1.7%) due to the crosslinking reaction, thereby improving the enzymatic degradation resistance.

SEM of these films after degradation are shown in Figure 6B. These films had different degraded surfaces. Compared with Figure 3A, all the surfaces of the non-crosslinked films (CSF, CSMCF1 and CSMCF2) and crosslinked films (CCSF, CCSMCF1, and CCSMCF2) became rougher and more uneven. Although CCSMCF2 was the smoothest before degradation, the surface developed bumps and hollows after the enzymatic degradation. In general, CCSMCF2 performed best from among these films.

### 3.5. Application on Fresh-Cut Wax Gourd

**Figure 7:**
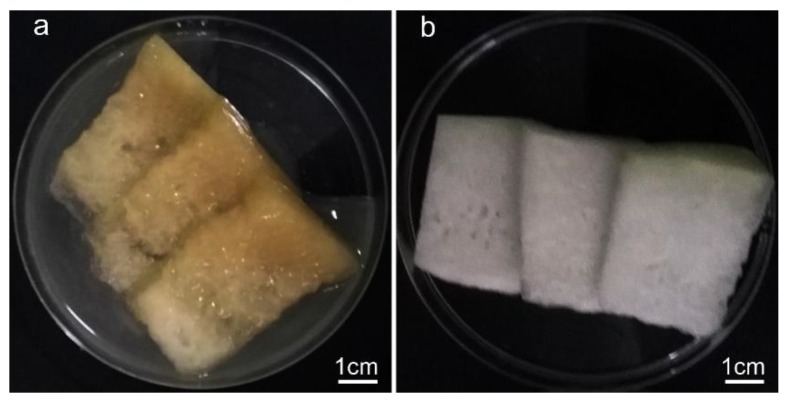
The storage effect on fresh-cut wax gourd after three days (**a**) without the prepared film and (**b**) with the obtained film CCSMCF2.

According to the comprehensive comparison, CCSMCF2 has low solubility, high tensile strength, excellent antibacterial activity, and low enzymatic degradation. Therefore, CCSMCF2, homogeneous, thin, and flexible, with no pores or cracks, was used for the preservation of fresh-cut wax gourd. After three days under room temperature, the preservative effects are illustrated in Figure 7. All treatments were observed by dryness, freshness, and color. The fresh-cut wax gourd in the control group demonstrated spoilage with much water and a yellow color, which was unacceptable to consumers. In contrast, the sensory quality of fresh-cut wax gourd treated with CCSMCF2 was acceptable. The samples showed a certain dryness, freshness, and excellent color, which indicated that CCSMCF2 was effective in maintaining the sensory quality of fresh-cut wax gourd. Similar results were also reported by other researchers, stating that chitosan-based films could prolong the shelf life of red grapes, beef, and white cheese [27].

## 4. Conclusions

The crosslinking process on films prepared from chitosan and methylcellulose by sodium trimetaphosphate proved to be an advantageous strategy to modify and improve the physicochemical properties. This technology is easy to use and has potential at an industrial level. The crosslinked films became more compact, flat, and smooth, due to the interactions among chitosan, methylcellulose, and sodium trimetaphosphate. The antibacterial activity of the crosslinked films remained high, and the examination of the stored films demonstrated that these films were promising for further application. In addition, the crosslinked films had low solubility and high tensile strength. After enzymatic degradation, the degradation ratios were low and the crosslinked films performed much better than the non-crosslinked films. Moreover, the obtained crosslinked film CSMCF2 maintained the sensory quality of fresh-cut wax gourd for three days at room temperature. Therefore, as an alternative to petroleum-based products, the crosslinked film using sodium trimetaphosphate will effectively prevent quality deterioration of food products and have potential in the food packaging field.

## Figures and Tables

**Table 1:** The different samples with different reaction parameters.

Projects	Parameters
Addition of methylcellulose	0, 30 wt %, 50 wt %, 70 wt %, and 100 wt %
STMP concentration	0.1 wt %, 0.2 wt %, 0.3 wt %, 0.4 wt %, and 0.5 wt %
Crosslinking time	0.5 h, 1.5 h, 2.5 h, 3.5 h, and 4.5 h

**Table 2:** Analysis of variance.

Source	Sum of Squares	df	Mean Square	F Value	*p*-Value Prob > F
Model	20.06	9	2.23	13.60	0.0012
A-MC/(CS + MC)	8.61	1	8.61	52.53	0.0002
B-STMPC	0.011	1	0.011	0.069	0.8009
C-CT	6.13	1	6.13	37.36	0.0005
AB	0.023	1	0.023	0.14	0.7220
AC	1.00	1	1.00	6.10	0.0428
BC	0.040	1	0.040	0.24	0.6364
A^2^	3.32	1	3.32	20.23	0.0028
B^2^	0.24	1	0.24	1.45	0.2678
C^2^	0.41	1	0.41	2.51	0.1573
Residual	1.15	7	0.16		
Lack of Fit	1.15	3	0.38		
Pure Error	0.000	4	0.000		
Cor Total	21.21	16			

CT = crosslinking time; STMPC = sodium trimetaphosphate concentration.

**Table 3:** The absorbance and color parameters of the developed films.

Films	A_500_	Color Parameters			
		L*	a*	b*	∆E
CSF	0.611	42.54	37.38	38.38	−
CSMCF1	4.375	56.31	23	59.55	29.06
CSMCF2	5.081	61.04	27.00	64.02	33.28
CCSF	5.472	79.81	−0.84	25.32	54.96
CCSMCF1	5.601	82.33	−1.6	12.77	61.31
CCSMCF2	5.632	86.21	−2.00	12.41	64.28

**Table 4:** Solubility, degradation ratio, and antibacterial properties of the obtained films.

Samples	Solubility (%)	Inhibition Ratio (%)		Degradation Ratio (%)
		*E. coli*	*S. aureus*	
CSF	1.5 ± 0.03	99.8	99.9	2 ± 0.17
CSMCF1	12 ± 0.11	99.1	99.6	3 ± 0.15
CSMCF2	23 ± 0.07	99.2	99.2	6 ± 0.19
CCSF	1 ± 0.05	99.6	99.7	1.8 ± 0.08
CCSMCF1	3 ± 0.09	99.1	99.0	1.7 ± 0.13
CCSMCF2	6 ± 0.12	99.0	99.0	1.7 ± 0.02
